# Strengths and challenges of longitudinal non-human primate neuroimaging

**DOI:** 10.1016/j.neuroimage.2021.118009

**Published:** 2021-08-01

**Authors:** Xiaowei Song, Pamela García-Saldivar, Nathan Kindred, Yujiang Wang, Hugo Merchant, Adrien Meguerditchian, Yihong Yang, Elliot A. Stein, Charles W. Bradberry, Suliann Ben Hamed, Hank P. Jedema, Colline Poirier

**Affiliations:** a Preclinical Pharmacology Section, Intramural Research Program, NIDA, NIH, Baltimore, MD 21224, USA; b Instituto de Neurobiología, UNAM, Campus Juriquilla. Boulevard Juriquilla No. 3001 Querétaro, Qro. 76230, México; c Biosciences Institute & Centre for Behaviour and Evolution, Faculty of Medical Sciences, Newcastle University, United Kingdom; d CNNP Lab (www.cnnp-lab.com), Interdisciplinary Complex Systems Group, School of Computing, Newcastle University, United Kingdom; e Laboratoire de Psychologie Cognitive, UMR7290, Université Aix-Marseille/CNRS, Institut Language, Communication and the Brain 13331 Marseille, France; f Neuroimaging Research Branch, Intramural Research Program, NIDA, NIH, Baltimore, MD 21224, USA; g Institut des Sciences Cognitives Marc Jeannerod, UMR 5229, Université de Lyon – CNRS, France

**Keywords:** Magnetic resonance imaging, Development, Ageing, Templates, Simulation, Non-human primate

## Abstract

Longitudinal non-human primate neuroimaging has the potential to greatly enhance our understanding of primate brain structure and function. Here we describe its specific strengths, compared to both cross-sectional non-human primate neuroimaging and longitudinal human neuroimaging, but also its associated challenges. We elaborate on factors guiding the use of different analytical tools, subject-specific versus age-specific templates for analyses, and issues related to statistical power.

## Highlights

• Strengths and challenges of longitudinal non-human primate MRI are described.
• Statistical power calculation of longitudinal and cross-sectional designs are provided.
• The impact of template choice on grey matter estimation is demonstrated.
• Recommendations for designing and analysing such studies are provided.

## 1 Introduction

Non-human primate (NHP) neuroimaging is a field progressively establishing itself as a crucial complement to human neuroimaging (Phillips et al., 2014; Roelfsema and Treue, 2014). The use of NHP models not only allows performing experiments that cannot be done in humans but also can shed light on the evolution of the primate brain. NHP neuroimaging has mostly been dominated by between-subject, cross-sectional experimental designs. However, within-subject, longitudinal designs are generally more powerful, but also more challenging. Here we compare longitudinal NHP neuroimaging with both cross-sectional NHP neuroimaging and longitudinal human neuroimaging. We describe its inherent strength in terms of statistical power and its specific strengths in developmental and ageing studies, as well as in interventional studies. We then describe specific challenges, encompassing data acquisition, image processing and statistical analyses.

## 2 Strengths of longitudinal approaches

### 2.1 Inherent strength: Cohort size and sample size

NHP neuroimaging operates within a stringent regulatory framework. While regulations vary between countries, a common principle applied globally is the ‘3Rs’ (*Replacement, Reduction and Refinement*) (Mitchell et al., 2021). As a consequence, animal numbers need to be kept to a minimum. Longitudinal designs present the crucial advantage of having greater statistical power than cross-sectional ones (Liu and Liang, 1997, Fitzmaurice et al., 2012, Tustison et al 2019), offering a powerful way to reduce the number of animals used.

**Fig. 1:**
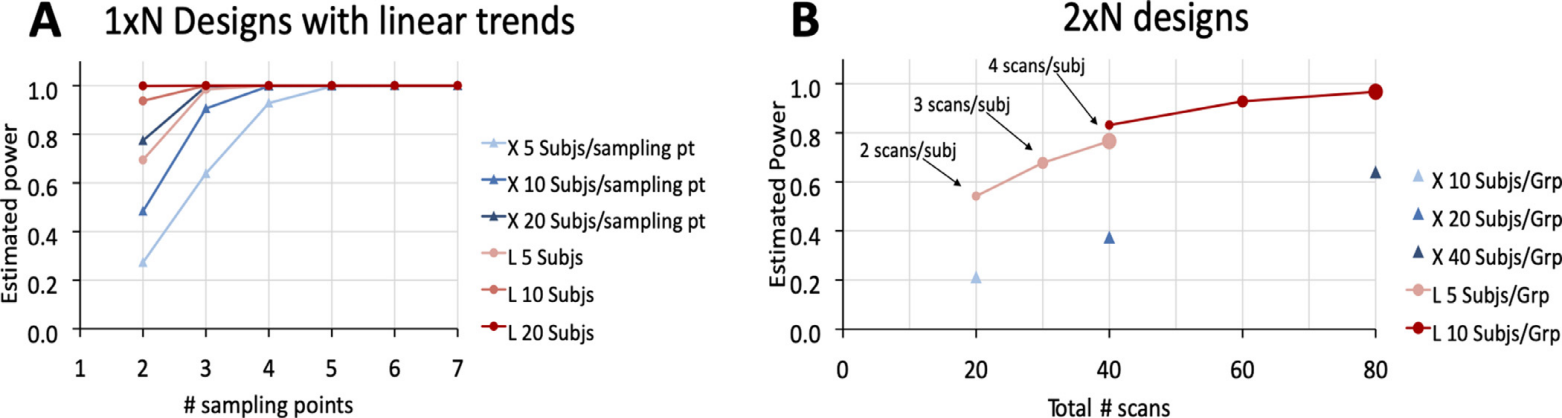
Power simulation of longitudinal (L) and cross-sectional (X) designs in two different scenarios: (A) a developmental/ageing study, where age varies within or between subjects; and (B) an interventional study with one control group and one treatment group (with equal number of subjects in each group). In the interventional scenario, the cross-sectional design with two groups is compared to a longitudinal design with two groups scanned once before treatment and once or several times after treatment (to study the sustainability of the treatment effect). The increased power for longitudinal experiments is illustrated using asymptotic statistical methods for simulated data with a fixed effect size and a fixed detection threshold, using the *longpower* package (https://cran.r-project.org/web/packages/longpower/index.html). It should be noted that practically, signal variability is not homogenous across brain regions and that different power value estimates would likely be obtained for different brain regions (Suckling et al 2014).

Statistical power, the probability to detect a true effect, depends on the size of the true effect, the statistical threshold, the sample size, and the amount of variability in the response variable. For a fixed effect size, fixed statistical threshold, and fixed sample size, statistical power therefore depends on the amount of variability in the response variable, with more variability leading to less power to detect statistical differences that can be attributed to the true effect. By focusing on within-subject differences, longitudinal studies circumvent the problem of inter-individual variability due to genes and gene X environment interactions, resulting in lower variability, and therefore, increased power. Importantly, longitudinal studies have greater statistical power than cross-sectional studies not only for a fixed number of subjects, but also for a fixed number of scans (Fig. 1). While longitudinal studies during childhood and adulthood both benefit from increased power (compared to a cross-sectional design), studies during adulthood benefit more, since inter-individual variability tends to be larger in adults than in juveniles (as individual gene X environment interactions increase with time).

Data sharing offers the opportunity to further increase the statistical power of longitudinal NHP studies by increasing the cohort size. The NHP neuroscience community has recently started to share neuroimaging datasets (Milham, 2018, 2020). While only cross-sectional datasets have been shared so far, the potential of longitudinal dataset sharing is even greater. Indeed, by focusing on within-subject effects, sharing of longitudinal data is less prone to problems linked to differences in data quality and scanning parameters between sites. An approach especially promising is the sharing of control group data in interventional studies to increase the sample size in developmental and ageing studies of healthy individuals. Another potential source of longitudinal neuroimaging datasets is the routinely acquired scans used to assess the health of NHPs (Basso et al., 2021; Balezeau et al., 2021). An increased sample size, combined with sharing of metadata (e.g. genetic information, early life history; for more details about metadata, see Poirier et al., 2021) will allow future investigation of individual differences in brain development and ageing.

### 2.2 Specific strength: developmental studies

Longitudinal MRI in NHPs is of considerable interest for investigating brain development from birth to adulthood or even prior to birth, thanks to foetal imaging. One major objective of such studies is to establish developmental trajectories in NHP species and to compare them across NHP species and with humans. Another goal is to develop experimental NHP models of human neurodevelopmental pathologies, thanks to either genetic mapping or pharmacological, environmental, or behavioural interventional approaches.

Several NHP longitudinal neurodevelopmental MRI databases have recently emerged (see Table 1). For instance, several rhesus macaque longitudinal MRI databases from postnatal to early adulthood can be found from different primate facilities mostly located in the USA (Scott et al. 2016; Young et al. 2017; Xia et al., 2020; Malkova, Heuer & Saunders 2006) but also in France (Rayson, et al., unpublished data) and in China (Liu et al., 2015), potentially describing distinct developmental trajectories of different sub-populations of rhesus macaques. Although less represented in comparison to rhesus macaques, similar longitudinal MRI brain data have also been collected in baboons, including early postnatal (e.g. Becker et al., 2021) and foetal MRI brain images (Kochunov et al. 2010; Liu et al., 2008, 2010), as well as in marmosets, from infancy to adulthood (Sawiak et al., 2018, Seki et al., 2017). Such in vivo non-invasive approaches include structural T1- and/or T2-weighted imaging (T1w and/or T2w) and to a lesser extent, Diffusion Tensor Imaging (DTI) or Resting State functional MRI (RS-fMRI). To our knowledge, no such longitudinal neurodevelopmental database exists for squirrel monkeys (*Saimiri sp.*) or mouse lemurs (*Microcebus sp.*), two genera of increasing relevance to the field of neuroscience (Fritz et al., 2020; Royo et al., 2021).

**Table 1:** Longitudinal brain development MRI databases in non-human primates.

Species	Centre	Publication	T1w/T2w	DTI	RS-fMRI	Initial cohort Size	Age Range
***Callithrix jacchus(marmoset, average lifespan, 5-7 years, max 12)***	*Brain Science Institute RIKEN, Wako, Japan*	*Seki et al., 2017*	*x*			*23*^a^	*1–30 months*
	*Cambridge University, UK*	*Sawiak et al. 2018*	*x*			*41*^a^	*3–27 months*
***Macaca mulatta(macaque, average lifespan, 15 years, max 35, gestation time, 168 days)***	*California National Primate Research Centre, USA*	*Scott et al. 2016*	*x*			*48*	*1–52 weeks*
	*University of Wisconsin, USA*	*Young et al. 2017**;**Xia et al., 2020*	*x*	*x*		*37*	*10–64 months*
	*National Institutes of Health, Bethesda, USA*	*Malkova et al., 2006*	*x*			*7*^a^	*1 week to 4 years*
	*CNRS centre, Lyon, France*	*Rayson, et al, unpublished*	*x*	*x*	*x*	*21*	*1.5 / 2.5 / 3.5 years*
	*Primate Centre of Academy of Sciences, Kunming, China*	*Liu et al., 2015*	*x*	*x*		*14*^a^	*6, 7, 8, 18, 16 months*
	*Yerkes National Primate Research Centre, USA*	*Shi et al., 2017**(Atlas)*	*x*	*x*		*40*^a^	*2 weeks, 3, 6, 12 months*
	*Oregon National Primate Research Centre, USA*	*Liu et al., 2020*	*x*	*x*		18*	*85, 110, 135 gestation days*
***Papio anubis(baboon, average lifespan, 25, max 37, gestation time, 26 weeks)***	*CNRS Primate Centre, Rousset, France*	*Becker et al., 2021*	*x*	*x*	*x*	*30*	*0-2; 8-10; 24-26 & 48 months*
	*Southwest National Primate Research Centre, USA*	*Kochunov et al. 2010*	*x*			*7*^a^	*17–25 gestation weeks*
	*Columbia University, New York, USA*	*Liu et al., 2008*, Liu et al., 2010	*x*			*5*^a^	*8–26 gestation weeks*

a Number of subjects scanned varies across time points.

Alongside these developmental longitudinal datasets, a handful of NHP developmental atlases exist. Regarding the macaque, Shi et al. (2017) have produced a developmental atlas from birth to 12 months of age, providing a parcellation of cortical and subcortical structures as well as brain fibre tract data. Liu et al. (2020) have recently issued an atlas of the foetal macaque brain at 85, 110 and 135 days of gestation, providing T2-based, fractional anisotropy (FA) and apparent diffusion coefficient (ADC) templates. These developmental macaque atlases can be co-registered to the multiple adult anatomical, histological and functional macaque atlases available to date. Regarding marmosets, Seki et al. (2017) and Sawiak et al. (2018) provide age-specific brain templates. In these latter age-specific brain templates, cortical brain regions are defined based on co-registration with adult marmoset histological (Majka et al., 2016) and anatomical sections (Paxinos et al., 2012). There is clearly a need for expansion of such atlases, covering longer macaque age ranges, and including other species such as baboons, squirrel monkeys, or mouse lemurs.

Using MRI in NHPs to study brain development provides critical benefits over human MRI datasets. First, while developmental trajectories in humans are well documented for healthy children aged 4 years old and up (Giedd et al., 1999; Sowell et al., 2004; Raznahan et al. 2012), it has been extremely challenging to collect high quality motion-free images from children below 4 years old. As a result, this has limited our knowledge about brain development in this very dynamic and critical age range. Few human studies have been able to scan longitudinally subjects below the age of two years old (Knickmeyer et al., 2008, Gilmore et al., 2012, Nie et al., 2014). This issue can be addressed when scanning nonhuman primate subjects, thanks to close anaesthesia monitoring, allowing one to obtain motion-free images at all age classes from birth into adulthood.

Second, as is the case for other types of longitudinal studies in humans, recruiting and following the same homogeneous infant cohort in order to obtain individual MRI images at several time-points remains a critical challenge, especially when the study is expected to cover a broad age range, for example from birth into adulthood. As a result, the majority of paediatric neuroimaging studies are cross-sectional, that is to say not necessarily tracking all subjects, at all time points (e.g., Evans, 2006, Jernigan et al., 2016, Van Essen et al., 2013). In NHPs, there are theoretically no such limitations, neither in terms of access to subjects, control of age homogeneity nor for the follow-up of intra-individual scanning at all predefined time points across development. The only limitation pertains to the capacity of reproduction in primate breeding centres, i.e. sample size.

Third, in order to secure the well-being of fully awake human infants and achieve motion-free brain images, the duration of data acquisition is often limited inside the MRI scanner, constraining the number of MRI multimodal sequences and their parameters (e.g. spatial resolution). In in-vivo NHP MRI acquisitions, securing the welfare and health of subjects by proper anaesthesia and physiological monitoring procedures under veterinary control allows a considerable increase in the time of a given MRI session. This provides unique opportunities for testing and developing more and much longer MRI sequences for better brain image quality and multimodal potentialities at this early stage of brain development. Additionally, this compensates for the decrease in the Signal-to-Noise Ratio of the MRI acquisitions incurred by the smaller NHP brain size (Kochunov and Duff Davis, 2010).

Fourth, there is now ample evidence that neurodevelopmental trajectories can be dramatically impacted by genetic (van Dyck et al., 2017) and non-genetic factors, thought to act via epigenetic mechanisms (Lester and Marsit, 2018). These non-genetic factors include social factors such as social deprivation during a so-called social critical period (Feldman, 2015; Sheridan and McLaughin, 2014) and physical factors such as sensory deprivation, anaesthesia (Disma et al., 2018), antipsychotic or analgesic maternal drug history (Hjorth et al., 2019, EUROmediCAT Steering Group, 2015, Nordeng et al., 2017), and metabolic and nutritional history (Barks et al., 2019). However, the precise mechanisms and factors by which neurodevelopment is impacted are still a matter of intense research. In this respect, NHPs represent a unique and extremely valuable model in which social and physical environmental factors as well as genetic and epigenetic mechanisms can be controlled or modified from conception into adulthood. Such experimental designs can thus be used for highly controlled interventional developmental studies in which a group of animals is submitted to a specific physical or social early rearing history experimental manipulation and its brain developmental trajectory is compared to the one of a normal control group.

### 2.3 Specific strength: ageing studies

The impact of ageing on brain structure and function is a topic of great interest which has been investigated mainly by a cross-sectional approach in humans and NHPs. Taking advantage of huge datasets and the development of algorithms to automatically analyse thousands of MRI scans, recent studies in humans have documented age-related brain changes consistent across subjects (Cole et al., 2018; Kaufmann et al., 2019; Madan and Kensinger, 2018; Walhovd et al., 2011; Wang et al., 2016). However, cross-sectional studies of ageing suffer from two main limitations which can be overcome by using a longitudinal approach. First, cross-sectional studies do not allow distinguishing true ageing effects from cohort effects. Because cross-sectional ageing studies compare brain scans from groups of subjects born in different years (different cohorts), they cannot distinguish true ageing effects from brain differences induced by factors unrelated to ageing that changed between cohorts’ birth years (e.g. nutrition of mothers during the prenatal period). Longitudinal studies allow the investigation of within-subject effects, which are immune to cohort effects, revealing true ageing effects. Secondly, cross-sectional studies, which can only reveal between-subject effects, treat individual differences unrelated to age as a source of noise. This approach is therefore ill-suited to investigate the source of individual differences in ageing effects. By capturing ageing processes in their within-subject effects, longitudinal studies allow for investigating individual variability by testing the interaction of between- and within-subject effects. To take full advantage of these methodological strengths, longitudinal studies must include multiple time points, covering the specific life period under investigation in each subject.

Despite these important advantages, longitudinal MRI studies are notoriously difficult to perform in humans. For pragmatic reasons, they can suffer from an important drop-out of participants and usually cover a short period of subjects’ lives (usually less than 10 years) (Fjell et al., 2017; Kuo et al., 2020; Scahill et al., 2003; Storve et al., 2014; Wu et al., 2013). NHPs therefore offer a unique opportunity to investigate true ageing effects in the primate brain. The fact that their longevity is shorter than humans (one year in a macaque life is equivalent to 3-4 years in a human life) allows researchers to collect data at a much faster speed and to cover longer periods of their lives (relative to their lifespan). In addition, given that NHPs spend their whole life in a controlled environment, it limits the sources of variation in brain function and structure over time which are unrelated to ageing.

### 2.4 Specific strength: interventional studies

The above paragraphs indicating the structural changes across the entire lifespan, underscore the critical need to control for age in longitudinal studies involving experimental manipulations such as disease models, rearing conditions, or training. Cross-sectional interventional studies in humans are limited by design, to comparisons between groups of subjects with different conditions, for example the comparison of a group having a certain disease with a “normal” control group. Between relatively large groups, such cross-sectional studies may be able to identify a difference between groups if the variance within groups is sufficiently small. If a between-group difference can be observed using a cross-sectional comparison (i.e. between a patient and a control group), the critical question remains whether these observed group differences were due to pre-existing traits or a consequence of the disorder (Jedema et al., 2021). Due to the fact that imaging data are not normally obtained prior to becoming a patient, this question remains very difficult to address in humans, although large prospective longitudinal human studies such as the ABCD study (Volkow et al., 2018) and the IMAGEN study (Mascarell Maricic et al., 2020) will help to address this issue. In substance use disorder studies, the question that arises with all cross-sectional studies is whether any observed difference in the drug abuse group is the consequence of prolonged drug use, or reflects a pre-existing condition that conferred greater vulnerability to drug use or even a combination of these factors.

In contrast to cross-sectional studies, longitudinal studies make within-subject comparisons, thereby reducing the impact of between-subject differences and nuisance variables. When combined with a proper age-matched control group, these studies control for any pre-existing conditions and permit causal attribution of the group by time interaction to the experimental manipulation. Given the similarity in brain structure, circuitry, and anatomical assignment of function as well as similarities in drug kinetics and adaptations with long term treatment, longitudinal NHP studies provide critically important preclinical data for complex human conditions. In addition, in longitudinal NHP imaging studies there is much better experimental control over the entire lifespan of the experimental and control groups compared to clinical populations (for example, substance abusers using a combination of multiple, different drugs versus a college student control group).

The value of NHPs to interventional longitudinal studies goes beyond preclinical studies and can serve to shed light on fundamental neuroscience research questions. For instance, NHPs can be trained to perform sophisticated cognitive tasks using operant conditioning techniques (Buffalo et al., 2019; Gámez et al., 2018). Macaques have been taught to categorize complex stimuli from all modalities (Freedman and Assad, 2016; Mendoza et al., 2018), to use abstract and dynamic rules to guide their behaviour (Miller and Cohen, 2001; Bartolo and Averbeck, 2020), to solve complex spatial cognition and game theory tasks (Georgopoulos et al., 1989; Crowe et al., 2005; Lee and Seo, 2016), and to learn to control brain machine interfaces with novel decoding requirements (Sadtler et al., 2014; Athalye et al., 2017; Golub et al., 2018). In addition, NHPs are an excellent model to obtain an evolutionary perspective on the neurobiology of cognitive traits originally considered human specific, such as language and music (Petkov and Ten Cate, 2019; Balezeau et al., 2020; Mendoza and Merchant, 2014; Merchant and Honing, 2014). Since the training periods in these elaborate tasks can take many months, the use of longitudinal imaging at different learning stages can reveal important structural changes in the grey and white matter of the underlying brain circuits. Because NHPs learn these complex tasks more slowly, there are more opportunities to scan them at different stages of learning and thus there is a better possibility to study the correlation between learning and structure. In contrast, because humans learn the complex tasks “too” quickly, they usually can only be scanned at stages before learning or after learning is complete.

Other applications of interventional longitudinal studies include investigating the impact of early rearing conditions onto brain development and cognition (Howell et al., 2019), as well as studying adult cortical plasticity following acute controlled brain damage or limb immobilization and subsequent rehabilitation or remediation strategies, whether pharmacological, behavioural, surgical or technological (e.g. brain-machine interfaces). The advent of genetically modified NHPs, whether marmosets (Kumita et al., 2019, Yoshimatsu et al., 2019) or macaques (Liu et al., 2016), opens the way to study gene control over both behaviour and brain structural and functional organization, in normal and pathological neurodevelopment, ageing and psychiatric/neurological disorder onset (Park and Silva, 2019). The goal is then to generalize multilevel or latent growth modelling approaches classically applied to the characterization of longitudinal brain trajectories (Mills and Tamnes, 2014) for comparison across the experimental and control groups.

## 3 Challenges of longitudinal MRI datasets

Despite numerous benefits, NHP longitudinal neuroimaging studies come with their own challenges. Some challenges are specific to particular applications, while others are generic. Here we describe what we consider to be the main challenges, related to experimental design, data acquisition, image processing, and statistical analyses.

### 3.1 Experimental design

Despite a reduction of the drop-out challenge characteristic of human longitudinal studies, NHP longitudinal studies are constrained by a limited number of subjects, for ethical (see Section 2.1) and pragmatic reasons. In discovery science, when the biologically relevant minimum effect size is not always known, power calculations are difficult to perform, making it difficult to justify exploratory studies involving large numbers of animals. The lack of universally accepted power calculators suitable for longitudinal designs only exacerbates this problem.

In addition, the number of available subjects is limited by the cost of NHP housing as well as the capacity of primate breeding centres. This last limitation is inflated in interventional neurodevelopment experiments which most often include, in addition to a test group, a control group to which the test group is compared. If the experimental intervention affects health parameters (e.g. increasing sensitivity to infections), the test group size might decrease with time. As a result, larger groups need to be provided at the beginning of the study, in anticipation of these losses. Growing evidence indicates that independent validation of the reported main effects is crucial when analysing longitudinal datasets (Herting et al., 2018). This can be done by multiple statistical approaches, such as independent tests on two independent subsamples of the data or leave one out or k-fold statistical cross validation procedures. The ability to perform such analyses is fully tied to cohort size.

For developmental studies, a related challenge is the ability to scan all individuals within the same age windows. This is especially critical in the very early months following birth in which brain morphology (in terms of myelination, overall brain size and gyrification) can change dramatically on a weekly basis. This can be complicated by the fact that births often happen at very short intervals in primate breeding centres, which in turn requires consistent access to the MRI facility. The presence of an MRI facility at the same location as the breeding centre tends to (partially) mitigate this problem.

### 3.2 Data acquisition

**Fig. 2:**
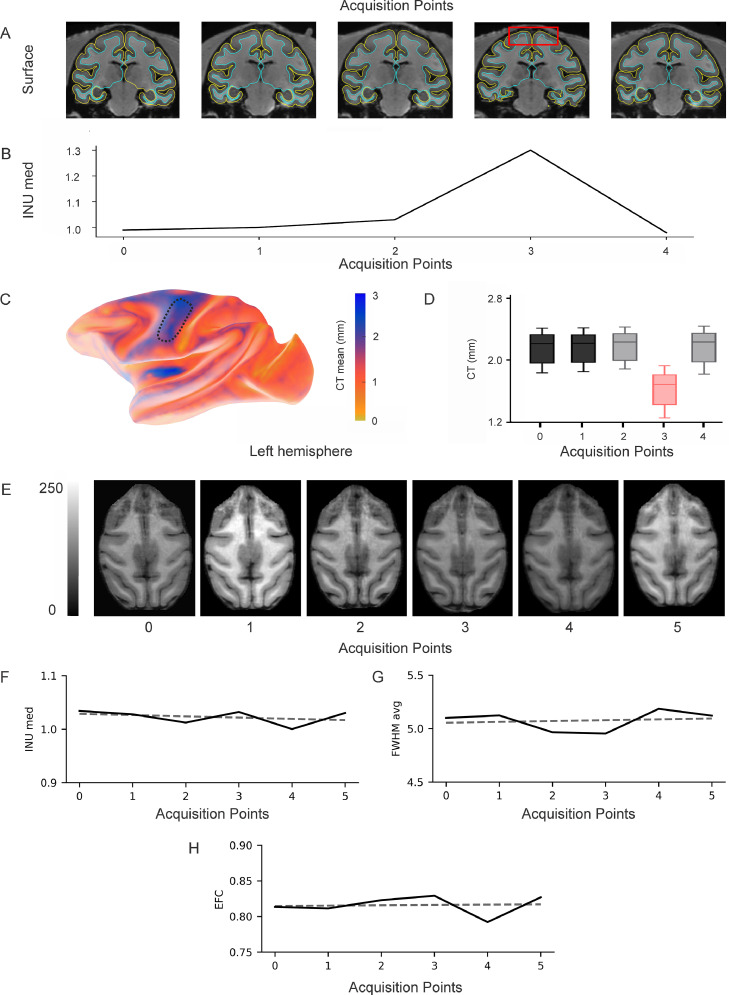
Quality control (QC) of T1w longitudinal images. A-D. Outlier identification. A. Brain surface estimation of an anesthetised macaque during five time points using PREEMACS Long. B. Estimation of intensity non-uniformity (INU) across acquisition points using MRIqc (Esteban et al., 2017) customized for the macaque by PREEMACS. C. Mean of the Brain Surface for the five acquisition points. The ROI defined by the dotted line corresponds to the precentral gyrus (pcg). Colour bar of cortical thickness (CT) in mm. D. CT estimation of pcg for each acquisition point. Point 3 is considered an outlier with a pcg CT and an INU that were statistically different from the other time points. E-H. Lack of longitudinal trends on QC metrics associated with head motion during awake scanning. E. Longitudinal scans of an awake macaque (one scan every 6 months) F. Median of INU field (INU med) as extracted by the N4ITK algorithm (values closer to 1.0 are better) across six acquisition points. G. Full-Width Half Maximum Smoothness (FWHM) of the spatial distribution of the image intensity values in units of voxels (lower values are better) for each time point. H. Entropy Focus Criterion (EFC) as a function of time points. EFC uses the Shannon entropy of voxel intensities as an indication of ghosting and blurring induced by head motion (lower values are better). The dotted line (F-H) corresponds to the best linear model between the QC metrics and the six acquisition points, with a slope close to zero for the three QC metrics, indicating no temporal trends in head motion.

All longitudinal NHP studies require hardware and software to be held constant during the whole experiment, which can sometimes last years. However, NHP neuroimaging approaches are often characterised by customized equipment and acquisition protocols in a state of constant refinement (Milham et al., 2018, 2020). The need for stability of longitudinal studies can therefore be in conflict with the drive for methodological and technical improvement, especially when imaging facilities and equipment are shared by multiple research groups. Moreover, to assure stable image quality, the head of the animal needs to be consistently positioned in the scanner field across scans and various custom radiofrequency coils for NHPs have been presented in recent years to help address this issue (Belcher et al., 2013, Gilbert et al., 2019, Quan et al., 2020). A potential risk is that experimenters become more proficient over time at positioning the heads of animals relative to the coils and the magnet, which can have an impact on image quality, especially when one uses non-standardized arrangements of surface coils. For awake NHP imaging, an additional problem is the impact of animals’ movements on image quality: if awake animals are not already well accustomed to the scanning environment and radio-frequency noise, the amplitude and frequency of their movements can decrease over time, resulting in a progressive improvement in data quality. It is therefore important to assess data quality *a posteriori*, and to identify outliers or check that data quality is not correlated with the longitudinal design (i.e. data quality increasing with time) (Fig. 2).

Another challenge specific to developmental studies in anaesthetised NHPs is the choice of the anaesthetic drugs. Anaesthesia allows minimizing head and body motion (which itself can alter the homogeneity of the magnetic field) and scanning for longer periods of time, thus making it possible to acquire multimodal imaging data. However, anaesthesia also has the potential to critically interfere with early brain development, due to the fact that most anaesthetics enhance GABAergic neurotransmission and depress NMDA neuromodulation, two key players of cortical plasticity. In addition, most anaesthetics show a high degree of neurotoxicity on repetitive exposure, including isoflurane, increasing apoptosis of neurons and oligodendrocytes in immature animals if administered within the critical neurodevelopmental period in which synaptogenesis is maximal (Brambrink et al., 2012; Coleman et al., 2017; Hays & Deshpande, 2011; Noguchi et al., 2017; Schenning et al., 2017). Dexmedetomidine, which provides an optimal trade-off between neurotoxicity and sedation, may be considered as sufficient for short scanning sessions in the youngest individuals (Koo et al., 2014) and has been successfully used to monitor resting state activity under anaesthesia (Fukuda et al., 2013; Brynildsen et al., 2017). In any case, it is recommended that the possible behavioural effects of repeated anaesthesia be tracked by an ethologist. Likewise, providing for a control group in which scanning (and thus anaesthesia) will only start after early childhood development is over should be envisioned. Whether scans on this control group should start before or after the important cortical changes associated with puberty is a matter for discussion. Alternatively, control groups could consist of independent individuals with no prior anaesthesia history, for each target age. The question of the interference of anaesthesia with neurodevelopmental trajectories thus turns out to be a scientific question in itself, of crucial impact on the neurodevelopmental longitudinal studies discussed here.

### 3.3 Image processing

#### 3.3.1 Analyses in population-based standard space

For many group analyses, MR images need to be moved into a generic population-based reference space via a co-registration process referred to as Normalization. Such transformation of original images can be performed using linear or non-linear deformation and allows for comparison of brain images at the voxel level. Normalization is also needed to co-register individual brains onto an atlas of interest (cytoarchitectonics, myeloarchitectonics, fiber tracking etc.), in order to identify comparable (cortical) areas across subjects.

A number of longitudinal anatomical data processing methods or pipelines have been proposed, including, but not limited to, Ashburner and Ridgway (2013), Reuter et al. (2012), Holland and Dale (2011), and Avants et al. (2010). The pipelines usually include two normalization steps between individual scans of different subjects taken at multiple time points: one normalization step from individual scans to a subject-or age-specific template, and a second normalization step from these templates to a final common reference space for NHPs (such as INIA-19, Rohlfing et al., 2012, NMT, Seidlitz et al. 2017), or a study specific average template that is independent of subject or age. The two normalization steps may iterate several times for the convergence of normalization parameters.

Longitudinal data processing aims to preserve within-subject changes for final statistical analyses. However, without special attention, many of the processing steps can introduce bias and influence the within-subject variance preservation. Several approaches for reducing bias have been introduced, such as symmetrical normalization to reduce interpolation bias (Avants et al., 2010 Holland and Dale, 2011; Nakamura et al., 2011; Reuter and Fischl, 2011) and building robust, unbiased, within-subject templates based on median or mean values and simultaneous co-registration of all time-points (Ashburner and Ridgway, 2013; Reuter et al., 2012).

Most non-linear registration procedures attempt to minimize the difference between intensity values of each individual scan and the study-based template with a wide range of degrees-of-freedom parameters. Compared to linear registration, this difference-minimization procedure can improve the registration, but it can also introduce bias of the individual scan toward the template, affecting the final statistical tests or the final detection sensitivity.

**Fig. 3:**
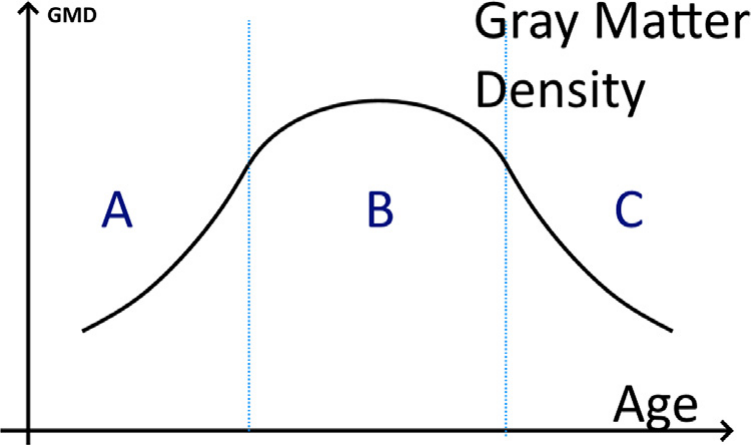
Anatomical measures such as Gray Matter Density (GMD), can change drastically across the lifespan, especially during early development (A) and late adulthood (C).

While there is general agreement that using a subject-specific template is best for longitudinal studies during adulthood (Iglesias et al., 2016; Reuter et al., 2012; Reuter and Fischl, 2011; Ashburner et al., 2013), no such consensus has been reached for developmental studies where both subject-specific and age-specific templates have been used in combination with different normalization procedures (Scott et al., 2016; Ball et al., 2019) to characterize the drastic changes during these more dynamic phases of the lifespan (Fig. 3).

**Fig. 4:**
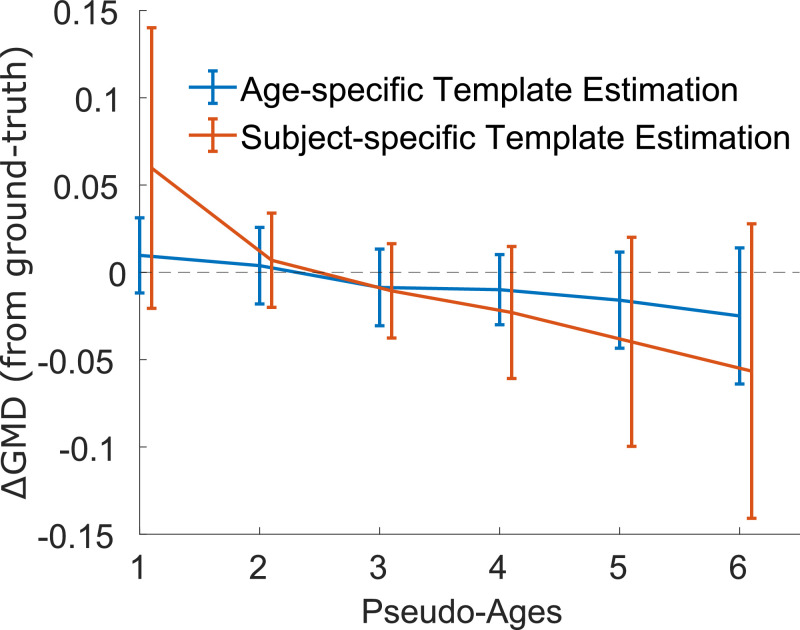
Simulation results showing the difference between grey matter density estimates and the ground truth following registration to an age-specific template (blue) and a subject-specific template (orange) in a pipeline combining linear and non-linear registration steps.

Here, we demonstrate using a simulation experiment of VBM data that the template choice is important when the pipeline contains both linear and non-linear registration steps to a final common space (see online Supplementary text). Specifically, the grey matter density (GMD) estimate after non-linear registration to the age-specific template is closer to the ground truth compared to the GMD estimate after non-linear registration to a subject-specific template (Fig. 4). In addition, the variance of the GMD estimate following registration to the subject specific template is larger. Given the greater deviation from the ground truth as well as the larger variance, the GMD estimate after registration to the age-specific template will have greater sensitivity to detect real changes during neurodevelopment. Similar results are expected in longitudinal studies during old age, a life period also characterized by notable brain changes (Fig. 3 phase C). In addition, our simulation demonstrates that the template choice does not seem to impact pipelines containing dual non-linear registrations steps to a final common template, although this may reduce overall sensitivity to detect real changes.

#### 3.3.2 Analyses in subject-specific space

Some analyses do not require images to be normalized into a generic population-based reference space. For instance, measures of whole brain volume, total amount of grey matter, regional cortical volume (e.g. temporal lobe volume, occipital lobe) and subcortical volume can be extracted for each subject at each time point (Mills and Tamnes, 2014) without voxel-to-voxel correspondence. Overall and regional cortical thickness, surface areas and gyrification measures (e.g. gyrification index) can also be assessed (Mills and Tamnes, 2014). In this case, images can be left in their native space, decreasing potential for biases. Nevertheless, for longitudinal studies during early and mid-adulthood, when within-subject changes over time are expected to be small (compared to between-subject differences), specific data processing can improve detection of longitudinal changes in native space. For instance, FreeSurfer longitudinal pipeline (Reuter et al., 2012) computes subject-specific tissue probability maps to improve the segmentation results, and subject-specific meshes to improve surface extraction.

#### 3.3.3 Limitations of human longitudinal pipelines

To date, longitudinal pipelines have been primarily developed for human MRI data. Such workflows include ANTs Longitudinal Cortical Thickness Pipeline (Tustison et al., 2017), FreeSurfer Longitudinal Pipeline (Reuter et al., 2012), and The Longitudinal registration and Longitudinal method (LL method) (Aubert-Broche, 2013). However, human pipelines directly applied to NHP data can generate errors at many steps (skull-stripping, subcortical labelling, segmentation of grey and white matter, particularly in visual and precentral cortex). These errors require manual intervention, which is time consuming and has the potential to bias the analysis. Another limitation of applying human pipelines is their primary focus on voxel-based or surface-based changes (for instance, FreeSurfer focuses on surface-based analyses, SPM and ANTs on voxel-based analyses). In NHP neuroimaging, both types of analyses are useful and ideally, a workflow for longitudinal NHP data would allow both. A new approach to automatically process cross-sectional and longitudinal NHP data is described elsewhere in this special issue (Garcia-Saldivar et al., 2021). Briefly, PREEMACS (pipeline for PREprocessing and Extraction of the MACaque brain Surface) is a set of tools taken from several image processing programs commonly used for human data and customized for rhesus monkey imaging, avoiding manual intervention. While this approach focuses on surface-based analyses, it should be possible to combine its pre-processing steps (including the automatic skull extraction and the debiasing approach) with voxel-based pipelines such as SPM or CAT (Gaser and Dahnke, 2016) to obtain voxel-based analyses without, or only minimal, manual intervention.

### 3.4 Statistical analyses

#### 3.4.1 Separating between- and within-subject effects

Independent of the strategy used to process images (cf. Section 3.2), longitudinal studies need to be analysed with statistical approaches specific to longitudinal designs, taking into account the dependency of repeated measures within subjects. In developmental and ageing studies, the null hypothesis is that there is no within-subject time effect (i.e. no age effect). In interventional studies, the null hypothesis is that within-subject effects are similar in different groups (i.e. no interaction between within-subject time effect and between-subject group effect). Testing within-subject effects can become an issue when the covariate of interest changes not only over time (i.e. within subjects) but also between subjects (within one group). Such a situation arises when researchers track age-related changes in subjects of different ages at the beginning of the study, a strategy often used to increase the sample size in ageing studies (e.g. Fjell et al., 2017; Storsve et al., 2014; Tamnes et al., 2017; Wu et al., 2013). In this specific case, it is important to separate within- from between-subject effects in the statistical analysis. Only within-subject effects will reveal true and accurate ageing effects, since between-subject effects are potentially biased by cohort effects (cf. Section 2.2) and by individual differences of genetic origin (especially when sample sizes are modest).

#### 3.4.2 Parameter estimation

The next issue relates to the estimation of the fixed within-subject effects parameters. Here we briefly review three popular statistical tools used to analyse longitudinal data: Linear Mixed Effects modelling (LME) (Chen et al., 2013), Sandwich Estimator (SwE) models (Guillaume et al., 2014), and Permutation Analysis for Linear Models (PALM) (Winkler et al., 2014, 2016).

*Linear mixed effects modelling (LME):* Linear mixed effects modelling can take into account multiple factors, including within-subject repeated measures factors, between-subject factors, or a mixture of both. LME has great flexibility in its modelling and estimation of the variance-covariance structures for both random effects and residuals. Traditional general linear models (such as ANOVA/ANCOVA) have much stricter assumptions on the variance-covariance structure and cannot model the covariance structure when there is random deviation of subjects at the levels of a random-effects factor. Due to the flexibility of LME modelling, it has been used extensively in a variety of experimental designs and for the description of all these various possibilities, that are outside the scope of this review, we refer the reader to detailed descriptions on LME modelling (Fitzmaurice et al., 2012, Chen et al. 2013, and Bates et al., 2015). Briefly, the response $y_{i}\in R^{N_{i}}$ (for subject *i*, and response vector length *N_i_*) can be described as the sum of fixed effect $\beta \in R^{P}$ and random effect $\gamma_{i}\in R^{Q}$, i.e., $y_{i}=X_{i}\beta +Z_{i}\gamma_{i}+\epsilon_{i}$ (with $X_{i}\in R^{N_{i}\times P}$ and $Z_{i}\in R^{N_{i}\times Q}$). The random effect $\gamma_{i}\;$ follows a normal distribution with variance $\Psi \in R^{Q\;\times \;Q}$, i.e., $\gamma_{i}∼N\left(0,\Psi \right),$ and a within-subject residual $\epsilon_{i}\in R^{N_{i}}$ following a normal distribution with variance $\Sigma_{i}\in R^{N_{i}\times N_{i}}$. Overall, the response vector is modelled as a multi-variate normal distribution, $y_{i}∼N\left(X_{i}\beta ,\;Z_{i}{\Psi Z}_{i}^{⊤}+\Sigma_{i}\right)$. By taking advantage of the available LME estimation package *lme4* (Bates et al., 2015), it is possible to deal with various variance-covariance structures for the between-subject Ψ matrix and the within-subject $\Sigma_{i}$ matrix, from the simplest diagonal matrix (i.e., no correlation among factors) to only requiring symmetry and positive definite. A method to use Akaike Information Criteria (AIC) or other criteria to select among multiple available LME variance-covariance matrix structures has been presented (Müller et al., 2013).

Practically for neuroimaging studies, 3dLME (Chen et al., 2013) and lme4 (Bates et al., 2015), can be used to model a variety of complex experimental designs and directly test the factor of interest without requiring implementation detail. For example, 3dLME allows the user just to specify the component of interest without building any design/contrast matrix. 3dLME can be applied in situations with a few response variables up to tens of thousands of voxels in voxel-wise fMRI datasets. However, such great modelling flexibility usually requires prior knowledge to choose certain models, which is not trivial. In addition, structural datasets with many voxels, as seen in typical high resolution NHP scans, can quickly exceed the capacity of even large computation clusters.

*Sandwich estimator (SwE)*: The Sandwich estimator (SwE) method focuses on population averages of longitudinal data and it has some adjustments for small sample size. SwE models implicitly handle within-subject covariance as opposed to LME models where users are required to explicitly specify or choose a variance-covariance matrix structure, and different random effects (such as including random subject-level intercepts/slopes or not), which is difficult in practice. From an optimization viewpoint, SwE separates within- and between-subject covariance such that the estimation can be as efficient as a generalized least square (GLS) method (thereby avoiding non-converging iterations as in the LME method).

Specifically, for the i^th^ individual (i=1,…,M) with $N_{i}$ observations, SwE models $y_{i}=X_{i}\beta +\epsilon_{i}^{*}$, in which $y_{i}\in R^{N_{i}}\;$contains $N_{i}\;$observations, $X_{i}\in R^{N_{i}\times P}$ is the design matrix, $\beta \in R^{P}$ denotes P fixed effects, and $\epsilon_{i}^{*}$ indicates an individual marginal error term. The fixed effects parameter β is estimated as $\hat{\beta }={\left(\sum_{i=1}^{M}X_{i}^{⊤}X_{i}\right)}^{-1}\sum_{i=1}^{M}X_{i}^{⊤}y_{i}$. Its covariance ($S\in R^{P\times P}$) is estimated as

$$\mathrm{var}\left\{\hat{\beta }\right\}=\underset{\text{Bread}}{\underset{︸}{{\left(\sum_{i=1}^{M}X_{i}^{⊤}X_{i}\right)}^{-1}}}\underset{\text{Meat}}{\underset{︸}{\left(\sum_{i=1}^{M}X_{i}^{⊤}{\hat{V}}_{i}X_{i}\right)}}\underset{\text{Bread}}{\underset{︸}{{\left(\sum_{i=1}^{M}X_{i}^{⊤}X_{i}\right)}^{-1}}},$$

in which $\hat{V_{i}}\in R^{N_{i}\times N_{i}}$ denotes the covariance of the i^th^ subject's fitting error/residual ($e_{i}=y_{i}-X_{i}\hat{\beta }$), i.e., $\hat{V_{i}}=e_{i}e_{i}^{⊤}$. Such subject-wise error covariance is chosen to be substituted with group-wise homogeneous error covariance estimation for each group, i.e., a group g(having subjects index set as $I_{g}$) share the same fitting error covariance ${\hat{V}}_{g}=\frac{1}{m}\sum_{i\in I_{g}}e_{i}e_{i}^{⊤}\;$. For the inference on the parameter $\hat{\beta }$, SwE uses a Wald test $T={\left(C\hat{\beta }\right)}^{⊤}{\left(\mathrm{CS}C^{⊤}\right)}^{-1}\left(C\hat{\beta }\right)/q\;$ for the null hypothesis $H_{0}:C\hat{\beta }=0$, in which qdenotes the rank of the contrast matrix $C\in R^{P\times P}$. All the estimations are based on maximum likelihood estimation which requires a large sample number. The Wald test statistic may have a heavier tail with small samples rather than the usual $X_{q}^{2}$ distribution. To adjust for small samples in the estimation, SwE can accommodate a correction factor for the raw residual $e_{i}$ before estimating ${\hat{V}}_{i\;}\;$but this requires the choice of an approximate statistic and F distribution as the null distribution: $\frac{v-q+1}{\mathrm{vq}}{\left(C\hat{\beta }\right)}^{⊤}{\left(\mathrm{CS}C^{⊤}\right)}^{-1}\left(C\hat{\beta }\right)∼F\left(q,v-q+1\right)$, in which v denotes a degree of freedom parameter to be estimated and is advised to use group-wise homogeneous estimation.

*Permutation Analysis of Linear Models (PALM):* The maximum likelihood estimators are asymptotical, require large samples, and assume certain distributions (usually Gaussian) in inference. These conditions are frequently not met, especially with small sample sizes generally seen in longitudinal NHP data. As a result, these methods might inflate the power or induce a higher false positive rate (FPR). Alternatively, permutation methods provide more precise control of the FPR. Among the permutation-based tools, PALM can accommodate longitudinal data with each subject as a whole permutation block. The precise control of FPR is the result of building the null distribution with many permutations. With certain distribution assumptions (such as Generalized Pareto Distribution), the required number of permutations can be reduced (500 permutations for a minimal *P*-value of 0.2%), compared to general permutation-based tests (~5000 permutations). Furthermore, comparisons between smaller numbers of subjects is possible. For example, there can be 252 permutations with a minimal *P*-value being 0.4%, with as few as 5 subjects for 2 groups with the assumption of different variances for 2 longitudinal time points.

In detail, given a linear model y=Xβ+Zγ+ϵ in which $y\in R^{M}$ is the observed data for Msubjects, $X\in R^{M\times P}\;$denotes the design matrix with regressors of interest, $Z\in R^{M\times Q}$ denotes the design matrix with nuisance regressors, $\beta \in R^{P},\gamma \in R^{Q}$ denotes corresponding regression coefficients of interest and nuisance, and $\epsilon \in R^{M}$ is the residue. The null hypothesis is that there is no difference for a given contrast (such as a contrast between the estimation and 0, or a contrast between two subsets for a comparison between 2 groups), i.e., $H_{0}:c^{⊤}\hat{\beta }=0$ for only one contrast $c\in R^{P}$, or $C^{⊤}\hat{\beta }=0$ for S contrasts matrix $C\in R^{P\times S}$ (1≤S≤P). The permutability or exchangeability is that under the null assumption, the joint probability distribution function for the involved variables is not different between the 2 contrasts and can be found through permutations.

Anatomical data, such as volume, density, and thickness, are all bounded numbers (i.e., there is a physical upper bound and lower bound), making it difficult to meet the infinite distribution value range assumption required for the SwE method. Combined with better control of the FPR, the use of PALM to approximate the distribution is preferred for small sample sizes over the assumption of a specific distribution as in SwE.

Advantages and disadvantages of the three statistical approaches are summarized in Table 2.

**Table 2:** Advantages and disadvantages of each approach.

	AFNI's 3dLME	SwE	PALM
Advantages	Most flexible and can deal with missing data. Intuitive on data input, no need for design matrix and contrast matrix.	Finer control in the model over within-subject variance for longitudinal design	Exact control over FPR, not dependent on specific distribution. Supports almost all neuroimaging file formats and classical multivariate inference such as multivariate analysis of variance (MANOVA) or multivariate analysis of covariance (MANCOVA), making joint analyses possible (Winkler et al., 2016, 2018).
Disadvantages	Anatomical measurements, which have a finite value range violate the assumption of infinity value range distribution. Voxel-wise calculations are computationally costly, often exceeding computing resources with high resolution structural scans. Flexible modelling makes model selection difficult. Asymptotic estimation requires large sample size.	Anatomical measurements, which have a finite value range violate the assumption of Infinity value range distribution. Even with small number correction, its maximum likelihood asymptotic estimation requires large sample size.	Requires careful design and contrast matrix for longitudinal studies. Permutations are computationally costly.

## 5 Conclusion

Longitudinal primate neuroimaging offers unique opportunities and promises to open exciting new research ventures. However, it is also associated with specific challenges. Some of these challenges can be circumvented but need to be anticipated. Considering the human and financial investment necessary to acquire longitudinal datasets, and in line with the ‘3Rs’, sharing such datasets should be encouraged. However, considering the associated dilution of primacy of data origination, which is important to investigators’ career progression, it should also be properly rewarded.

## Data and Code Availability Statement

Data were used for illustration purpose only. The simulation code is available here: https://github.com/dawnsong/longitudinalVBMTemplateBias

## CRediT authorship contribution statement

**Xiaowei Song:** Formal analysis, Investigation, Writing – original draft. **Pamela García-Saldivar:** Formal analysis, Visualization, Writing – original draft, Writing – review & editing. **Nathan Kindred:** Visualization, Writing – review & editing. **Yujiang Wang:** Writing – review & editing. **Hugo Merchant:** Visualization, Writing – original draft, Writing – review & editing. **Adrien Meguerditchian:** Writing – original draft. **Yihong Yang:** Writing – review & editing. **Elliot A. Stein:** Writing – review & editing. **Charles W. Bradberry:** Writing – review & editing. **Suliann Ben Hamed:** Writing – original draft, Writing – review & editing. **Hank P. Jedema:** Writing – original draft, Writing – review & editing. **Colline Poirier:** Writing – original draft, Writing – review & editing.

## Declaration of Competing Interest

The authors declare no competing interests.

## Appendix Supplementary materials

Image, application 1

## Footnotes

Supplementary material associated with this article can be found, in the online version, at doi:10.1016/j.neuroimage.2021.118009.
