# Supplementary material for: Strengths and challenges of longitudinal non-human primate neuroimaging
Source: Neuroimage. 2021 Aug 1;236:118009. doi: 10.1016/j.neuroimage.2021.118009 (PMC8270888; doi:10.1016/j.neuroimage.2021.118009)
Supplement: Supplementary file 1 [file mmc1.docx]

Supplementary Information

# Different templates can bias the final estimation

In order to align individual scans to a common space, longitudinal processing pipelines typically use several steps of linear and/or non-linear registration to allow final voxel-wise comparison across all scans. Although pipelines can use multiple iterations of alignment, in its simplest form they use one linear or non-linear alignment step from an individual scan to either an age- or subject-specific template and a second linear or non-linear alignment step from this age or subject-specific template to a final common space (Fig S1). Non-linear registration procedures may achieve better alignment than rigid or affine transformations using a wide range of parameters. However, because the non-linear procedures attempt to minimize the difference between the input image and the template, they can introduce bias toward the reference template, thereby affecting subsequent statistical tests or the detection sensitivity (Avants et al 2010, Reuter et al 2012).


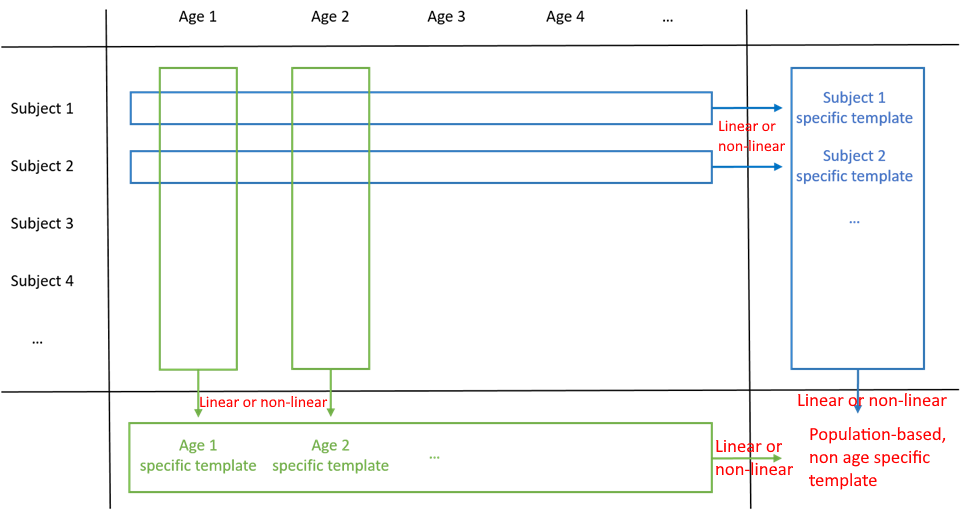


Figure S1. There are different combinations to register original scans to final common space.

As described in the main text, for developmental longitudinal studies, different strategies have been used to align individual scans to a final common space. Scott et al. (2016) used linear alignment of each individual scan to an age-specific template, which was subsequently non-linearly aligned to the population average template of the study. In contrast, Ball et al. (2019) adopted non-linear alignment of each individual scan to a subject-specific template, which was subsequently non-linearly aligned to the “National Institute of Mental Health Macaque Template” (NMT, Seidlitz et al., 2017). Consequently, these pipelines differed in the initial template that individual images were aligned to and the alignment method that was used (linear or non-linear), as well as the final common space in which the results were reported.

In the following simulation experiment of voxel-based morphometry (VBM) data, we demonstrate that the template choice is important when the pipeline contains linear and non-linear registration steps to a final common space (while focusing on a single iteration of alignments for simplicity). The template choice does not seem to impact pipelines containing dual non-linear registrations steps, but this may reduce overall sensitivity to detect real change.


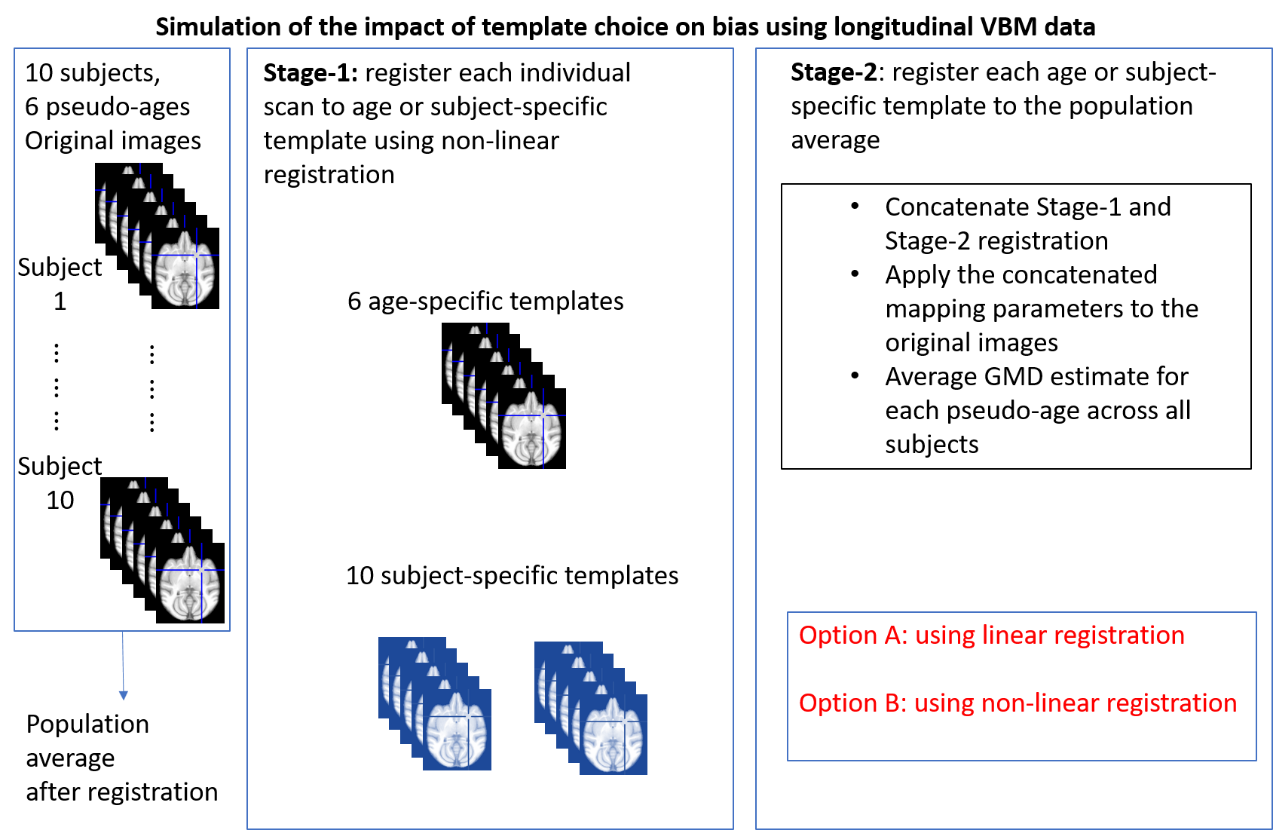


Figure S2. Schematic overview of the simulation.

## Possible estimation bias using different templates

### Theoretical analysis

To determine an optimal strategy for developmental studies, we performed a simulation experiment of longitudinal VBM data (Fig S2), testing the hypothesis that registration to different templates can affect the gray matter density (GMD) estimates.

Let’s assume that the GMD X*_an_* for the *n*^th^ subject at age *a* can be represented by the sum of the age-specific GMD (fixed effect *I_a_*), and the independent, random effects of age (*ε_a_*)and subject (*ε_n_*; random subject error includes measurement error), *X_an_* = *I_a_* + *ε_a_* + *ε_n_*. In addition, let’s assume *f_R_: X → Y* reflects the non-linear registration of an individual GMD measurement *X* to the reference template R. Then we have the registered image *Y = f (X ; R, Θ)* in the reference template space (*R*), where *Θ* denotes all related parameters involved in the non-linear registration to minimize the difference between *Y* and *R.*

Building a subject-specific template across all $A$ages before non-linear registration is essentially obtaining the expected value of GMD over the random variable of age, i.e., *X_n_* = *E_a_* { *X_an_* } = *E_a_* { *I_a_* + *ε_a_* + *ε_n_* } $\approx\left( \sum_{a=1}^{A} I_{a} \right) /A+{\epsilon_{n}=I}_{n}+\epsilon_{n}$which reflects the mean GMD for all longitudinal measurements for the *n*^th^ subject, including a subject-specific intensity term which is constant across ages, and an error term for this subject.

Building an age-specific template across all *N* subjects is essentially obtaining the expected value of GMD over the random variable of subject, i.e., *X_a_* = *E_n_* { *X_an_* } = *E_n_* { *I_a_* + *ε_a_* + *ε_n_* } $\approx$ *I_a_* + *ε_a_*, which includes an age-specific intensity term which is constant across subjects, and an error term for age *a*. After the non-linear registration, we would like to test the null hypothesis that the GMD does not change with age, or the average GMD over all subjects for each age, i.e., $Y_{a}=E_{n}\{Y_{an}\}\approx\sum_{n=1}^{N} Y_{an}/N$, is not related to age.

Note that the differences between *X_n_* and *X_a_* include the random error term and a different intensity term. When *X_n_* and *X_a_* values approach each other, the difference between age and subject-based template will be close to zero, and the two templates perform similarly without difference in the final estimation. Because developmental studies often span years during which the brain changes drastically (see main text), we expect the age-specific template, and consequently the registered image $Y$, to be closer to the ground truth (*I_a_*).

### Simulation

Based on the theoretical analysis above, we performed a simulation of GMD values from the left putamen region of interest (ROI) of the INIA-19 rhesus macaque template (Rohlfing et al, 2012). In addition, we chose the uniform, random variables *ε_a_* _~_ [-0.1,0.1] *and ε_n_* _~_ [-0.1,0.1] because the GMD or anatomical measurements have limited range values (0 ≤ GMD ≤ 1). In this simplified simulation, changes only occur in signal intensity within the ROI, the signal intensity of voxels within the ROI are independent of each other, and random effects of age and subject (which includes all other random factors) are assumed to be independent. In addition, our simulation assumes that optimal linear alignment can be achieved. Finally, please note that there is no manipulation of any parameters of the linear or non-linear registration steps.

For all voxels of the ROI (4279 voxels, 0.6mm^3^), we generated GMD values around 6 intensity levels *I_a_* (0.1 ≤ *I_a_* ≤ 1 with step size 0.18) for 10 subjects, and treated these intensity levels as pseudo-age points in a simulation for GMD changes during early development (main text Fig 3 phase A) (Morita et al., 2016). The GMD values averaged across all subjects at each age reflects the ground-truth GMD, *Î_a_*. The simulation for 6 intensity levels for all 10 subjects was repeated a total of 10 times. As can be seen (Fig S3A), the average GMD estimate *Y_a_^(a)^* after non-linear registration to the age-specific template is closer to the ground truth (*Y_a_^(a)^* – *Î_a_* blue line) than the average GMD estimate *Y_a_^(s)^*, after non-linear registration to the subject-specific template (*Y_a_^(s)^* – *Î_a_* , orange line). In addition, the variance of the GMD estimate following registration to the subject specific template is larger. When linear alignment is used for alignment to the final common template space (Fig S2: Stage 2, Option 2A), the greater deviation from the ground truth of GMD (as well as the larger variance) impacts the final GMD estimates. Consequently, the GMD estimate after registration to the age-specific template will have greater sensitivity to detect real changes during neurodevelopment (Fig S3A). In contrast, if the final alignment step to the common space is also non-linear (Fig S2: Stage 2, Option 2B), the GMD estimate in the final common space is not impacted by the choice of the initial template (age- or subject-specific templates; Fig S3B). Even though alignments to either the age- or subject-specific templates produce similar GMD estimates in the final common space, the difference minimization procedure of the second non-linear registration step increases the deviation from the ground truth. The non-linear registration tool in the present analysis uses FSL FNIRT (Andersson et al 2007), but similar results would be achieved using any other intensity based non-linear registration methods.


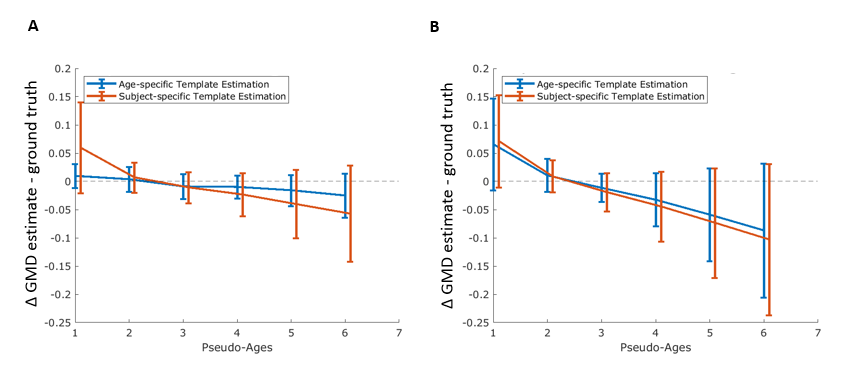


Figure S3 **A.** Simulation results showing the difference between gray matter density estimates and the ground truth following registration to an age-specific template (blue) and a subject-specific template (orange) in a pipeline combining linear and non-linear registration steps (Fig S2, Option 2A). **B.** Alignments to both age- and subject-specific templates produce similar GMD estimates in the final common space after two non-linear alignment steps (Fig S2, Option 2B).

**References Supplement**

- Andersson, J.L., Jenkinson, M., Smith, S., 2007. Non-linear registration aka Spatial normalisation FMRIB Technical Report TR07JA2. FMRIB Analysis Group of the University of Oxford.
- Avants, B., Cook, P.A., McMillan, C., Grossman, M., Tustison, N.J., Zheng, Y., Gee, J.C., 2010. Sparse Unbiased Analysis of Anatomical Variance in Longitudinal Imaging, in: Jiang, T., Navab, N., Pluim, J.P.W., Viergever, M.A. (Eds.), Medical Image Computing and Computer-Assisted Intervention – MICCAI 2010, Lecture Notes in Computer Science. Springer, Berlin, Heidelberg, pp. 324–331. <https://doi.org/10.1007/978-3-642-15705-9_40>
- Ball, G., & Seal, M. L., 2019. Individual variation in longitudinal postnatal development of the primate brain. Brain Struct. Funct. 224 (3), 1185–1201.<https://doi.org/10.1007/s00429-019-01829-5>
- Morita, T., Asada, M., & Naito, E., 2016. Contribution of Neuroimaging Studies to Understanding Development of Human Cognitive Brain Functions. Front. Hum. Neurosci. 10, 464.<https://doi.org/10.3389/fnhum.2016.00464>
- Reuter, M., Schmansky, N. J., Rosas, H. D., & Fischl, B., 2012. Within-subject template estimation for unbiased longitudinal image analysis. Neuroimage 61 (4), 1402–1418.<https://doi.org/10.1016/j.neuroimage.2012.02.084>
- Rohlfing, T., Kroenke, C.D., Sullivan, E.V., Bowden, D.M., Grant, K.A., 2012. The INIA19 template and NeuroMaps atlas for primate brain image parcellation and spatial normalization. Front. Neuroinform. 6, 27.<https://doi.org/10.3389/fninf.2012.00027>
- Scott, J. A., Grayson, D., Fletcher, E., Lee, A., Bauman, M. D., Schumann, C. M., Buonocore, M. H., & Amaral, D. G., 2016. Longitudinal analysis of the developing rhesus monkey brain using magnetic resonance imaging: birth to adulthood. Brain Struct. Funct. 221 (5), 2847–2871.<https://doi.org/10.1007/s00429-015-1076-x>
- Seidlitz, J., Sponheim, C., Glen, D., Ye, F.Q., Saleem, K.S., Leopold, D.A., Ungerleider, L., Messinger, A., 2017. A population MRI brain template and analysis tools for the macaque. NeuroImage. <https://doi.org/10.1016/j.neuroimage.2017.04.063>
